# Cytosolic Irradiation of Femtosecond Laser Induces Mitochondria-dependent Apoptosis-like Cell Death via Intrinsic Reactive Oxygen Cascades

**DOI:** 10.1038/srep08231

**Published:** 2015-02-04

**Authors:** Jonghee Yoon, Seung-wook Ryu, Seunghee Lee, Chulhee Choi

**Affiliations:** 1 Department of Bio and Brain Engineering, KAIST, Daejeon, Korea; 2 KAIST Institute for Optical Science and Technology, KAIST, Daejeon, Korea; 3 KAIST Institute for the BioCentury, KAIST, Daejeon, Korea

## Abstract

High-intensity femtosecond lasers have recently been used to irreversibly disrupt nanoscale structures, such as intracellular organelles, and to modify biological functions in a reversible manner: so-called nanosurgery and biophotomodulation. Femtosecond laser pulses above the threshold intensity sufficient for reversible biophotomodulation can cause irreversible changes in the irradiated cell, eventually leading to cell death. Here, we demonstrated that cytosolic irradiation with a femtosecond laser produced intrinsic cascades of reactive oxygen species (ROS), which led to rapid apoptosis-like cell death via a caspase and poly (ADP-ribose) polymerase 1 (PARP-1) signaling pathway. We further showed that cells with enhanced mitochondrial fusion activity are more resilient to laser-induced stress compared to those with enforced mitochondrial fission. Taken together, these findings provide fundamental insight into how optical stimulation intervenes in intrinsic cellular signaling pathways and functions.

Ultrashort-pulsed lasers including femtosecond-pulsed lasers can have greatly high intensity with relatively low average energy because of the extremely short pulse duration. Focused irradiation of high-intensity femtosecond laser can induce non-linear optical effects, such as multiphoton absorption and frequency doubling. As these non-linear phenomena can only occur in the tightly focused area, this reduces out-of-focus signals and has been applied in the field of biomedical imaging as multiphoton microscopy^1^. Recently, it has been reported that femtosecond laser pulses can be used to regulate biological functions, such as muscle contraction^2^,^3^, blood-brain barrier permeabilization^4^, cellular activation^5^,^6^, and gene transfection^7^, which is known as reversible biophotomodulation. Femtosecond laser stimulation within specific energy windows has been shown to induce the production of free electrons, also known as low-density plasmas, which can elevate intracellular Ca^2+^ levels^8^ or transiently disrupt the integrity of the plasma membrane^7^.

A high-intensity focused femtosecond laser pulses can induce highly reactive oxygen radicals also known as reactive oxygen species (ROS) in biological samples^8^,^9^. ROS are involved in multiple cellular signaling pathways as well as various pathophysiological processes^10^. Most intracellular ROS are generated as byproducts of oxidative phosphorylation in the mitochondria, which play a vital role in activation of intrinsic cell death process by releasing proapoptotic proteins^11^. Although laser-induced ROS regulate biological function in a reversible manner^1^,^3^, they are also related to the laser-induced cytotoxicity^9^,^12^. The exact molecular mechanisms by which optical stimulation may induce cytotoxicity remain unclear, although membrane disruption has been proposed as one possibility.

Our group has recently been engaged in the development of new optical methods that can be used for reversible modulation of biological functions by utilizing femtosecond-pulsed lasers. We have demonstrated that laser-induced photobiomodulation can be mediated by laser-induced intracellular ROS^1^,^3^. We reported previously that femtosecond laser stimulation induces two distinctive responses in primary cultured smooth muscle cells, i.e., reversible and irreversible responses, depending on the energy delivered^3^. In the present study, focused femtosecond laser stimulation on the cytosolic area induced marked fragmentation of the mitochondrial network, membrane bleb formation, and rapid retraction of the plasma membrane, leading eventually to apoptosis-like cell death. We further showed that the intrinsic signaling molecules caspase family and poly (ADP-ribose) polymerase 1 (PARP-1) are involved in laser-induced cell death.

## Results

### Femtosecond laser pulses induce irreversible changes in irradiated cells

**Figure 1:**
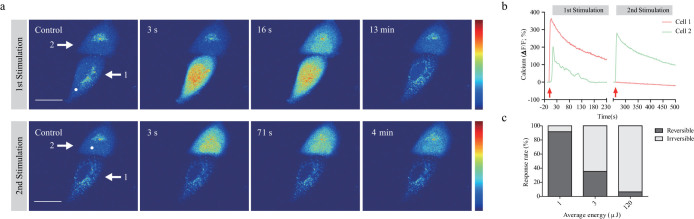
Laser-induced Ca^2+^ response and irreversible changes in HeLa cells. (a) Representative temporal dynamics of laser-induced Ca^2+^ response and cell death in HeLa cells. The white spot indicates the irradiated region. The numbers above each image indicate the time after laser stimulation at 0 s. Scale bar, 20 μm. (b) Quantification of laser-induced Ca^2+^ dynamics in the irradiated HeLa cell and the adjacent cell. Numbers in the baseline images in (a) indicate corresponding cells. (c) Deposited energy-dependent cell response rates. Irradiation time was varied from 0.96 to 121.83 μs. Laser output power was fixed at 1 W.

We investigated the mechanisms underlying the irreversible cytotoxic effects of femtosecond-pulsed laser irradiation using human epithelial carcinoma HeLa cells. As cellular responses to laser stimulation are mainly dependent on the irradiation laser energy, we fixed the laser output power at 1 W and observed cellular responses while changing the laser irradiation time from 1.96 to 196.83 μs. A femtosecond-pulsed laser was focused on ≤1 μm^2^ of the cytosolic area, and the evoked intracellular Ca^2+^ signal was measured as a readout in the irradiated cell. Transient increases in Ca^2+^ level were reproducibly induced by repetitive laser stimulation, while repetitive Ca^2+^ waves were not observed in cells showing typical irreversible changes (Fig. 1a). After the initial wave of laser-induced intracellular Ca^2+^ signal returned to the basal level, we irradiated the adjacent cell with the same optical parameters as used in the first stimulation. We previously found that laser-induced Ca^2+^ increases can be propagated to neighboring cells via gap junctions^3^. The second laser stimulation induced another Ca^2+^ increase in the irradiated cells; however, the Ca^2+^ wave did not propagate to the cell that was initially irradiated with high-energy laser and showed an irreversible response (Fig. 1b). The majority of responses were reversible in optical stimulation with an energy of 1 μJ, while the irradiation energy above 3 μJ caused mostly irreversible responses (Fig. 1c). These results collectively indicate that femtosecond laser pulses above a certain threshold can induce irreversible cytotoxic effects in the irradiated cells, resulting in non-responsiveness to further laser stimulation.

### Morphological characteristics of irradiated cells showing irreversible changes

**Figure 2:**
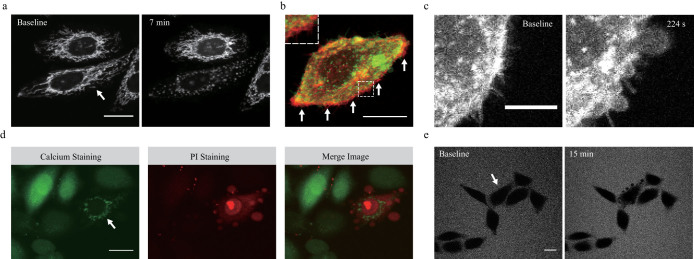
Morphological characteristics of laser-induced cell death. (a) Mitochondrial fragmentation after laser stimulation. The white arrow indicates the irradiated cell. (b) Plasma membrane shrinkage after laser stimulation in the irradiated HeLa cell. The red fluorescence indicates the baseline plasma membrane boundary and the green fluorescence indicates the altered boundary of the plasma membrane after laser irradiation. The white arrows indicate the region of plasma membrane retraction. The square outline with white dashed lines located at the top left shows a higher magnification image of the region of the square with white dashed lines located in the middle. Scale bar, 20 μm. (c) Membrane blebbing after laser irradiation. The numbers above each image indicate the time after laser stimulation at 0 s. Scale bar, 10 μm. (d) PI staining image of the irradiated cell 15 min after laser stimulation. The white arrow indicates the irradiated cell. Scale bar, 20 μm. (e) Intact plasma membrane of the irradiated HeLa cell. Gray signals outside the cells are fluorescence signals of 2 MDa-FITC dextran. The white arrow indicates the irradiated cell. Scale bar, 20 μm.

One of the hallmarks of irreversible changes was the punctuated pattern of fluorescent Ca^2+^ indicator in the cytosol. When cells were irradiated with lasers in the irreversible response range, the fluorescence aggregated in the intracellular membranous structures within a few minutes after laser stimulation (Supplementary Fig. 1a). As mitochondria play essential roles in intracellular Ca^2+^ regulation^13^, we confirmed that aggregated fluorescence was not due to selective Ca^2+^ uptake into the mitochondria by comparing mitochondrial morphology and Ca^2+^ indicator signals (Supplementary Fig. 1a). We found that mitochondria were significantly fragmented in the cells showing an irreversible response (Fig. 2a and Supplementary Movie 1). Immediately after laser stimulation, mitochondrial fragmentation was initiated from the vicinity of the irradiated region to the whole cytosolic area in cells showing irreversible responses (Supplementary Fig. 1b). Considerable levels of mitochondria-specific indicators leaked into the cytosol in cells showing irreversible responses along with extensive mitochondrial fragmentation (Supplementary Fig. 1c). Such leakage may reflect the loss of mitochondrial membrane potential, suggesting that laser stimulation can affect the morphology and function of mitochondria.

In addition to changes in the mitochondria, rapid shrinkage of the plasma membrane was also observed in cells showing irreversible responses. Plasma membranes stained with the plasma membrane indicator CellMask showed rapid retraction within 3 min after laser stimulation (Fig. 2b and Supplementary Movie 2). Moreover, membrane bleb formation was also observed during cell shrinkage (Fig. 2c). Mitochondrial potential loss, membrane retraction, and bleb formation are typical phenotypic characteristics of apoptosis^14^, and therefore we further investigated cell viability by adding propidium iodide (PI) to the culture medium. Only cells showing irreversible responses were stained strongly with PI (Fig. 2d). To confirm whether plasma membrane integrity is maintained throughout laser-induced cell death, 2 MDa-FITC dextran was added to the culture medium. The extracellular region showed bright fluorescence, and a distinct cell boundary was clearly observed on confocal microscopy (Fig. 2e). Membrane blebs were also negatively stained with 2 MDa-FITC dextran within 15 min after laser stimulation (Fig. 2e). The hydrodynamic size of 2 MDa-FITC dextran is 50 nm and that of the PI molecule is 5 nm. Therefore, these results indicate that the laser-induced irreversible response was accompanied by minimal loss of plasma membrane integrity in the irradiated cells (less than 50 nm). Next, we investigated whether laser-induced cell death has any deleterious effects on adjacent cells (Supplementary Fig. 2a,b). Although the irradiated cells shrunk and eventually died, adjacent cells showed normal patterns of growth and division. These observations indicate that femtosecond laser pulses induced highly selective irreversible responses limited only to the irradiated cells. We also found that femtosecond laser irradiation induced comparable cell death in various primary cells and cancer cell lines (Supplementary Fig. 3).

### Involvement of intrinsic ROS and signaling molecules in laser-induced cell death

**Figure 3:**
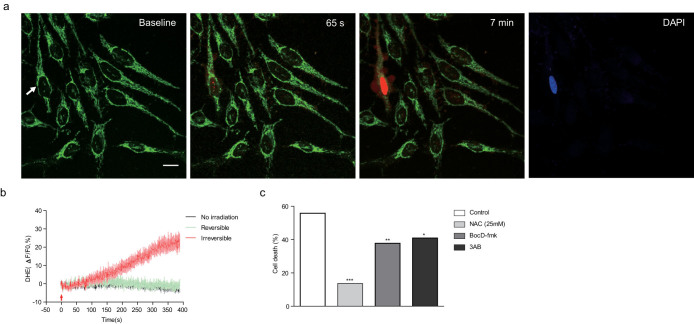
Laser-induced formation of intrinsic ROS. (a) Representative temporal dynamics of laser-induced intrinsic ROS formation in the irradiated cell. Green fluorescence indicates mitochondrial morphology stained by MitoTracker Green. Red fluorescence indicates intrinsic ROS stained with DHE. The white arrow indicates the irradiated cell. The numbers above each image indicate the time after laser stimulation at 0 s. Scale bar, 20 μm. (b) Quantification of laser-induced intrinsic ROS formation in the irradiated cell. The red arrow indicates laser irradiation. (c) Effects of chemical inhibitors against laser-induced cell death. HeLa cells were treated with NAC (antioxidant, 25 mmol/L), 3AB (PARP-1 inhibitor, 10 mmol/L), and BocD-fmk (caspase family inhibitor, 20 μmol/L), Experiments revealed the cell death ratio caused by laser stimulation (*n* ≥ 100 cells). *P < 0.5, **P < 0.01, ***P < 0.001 (Chi-square test).

We have previously reported that laser-induced ROS were involved in the increases in intracellular Ca^2+^ levels following laser irradiation^3^. High concentrations of intracellular Ca^2+^ are also known to induce cytotoxicity^15^. However, we found that inhibition of ryanodine receptors located on the endoplasmic reticulum using a pharmacological receptor antagonist ryanodine (EMD4Biosciences, USA), had little effect on laser-induced cell death, although the same treatment significantly blocked the laser-induced increase in intracellular Ca^2+^ level (Supplementary Fig. 4a,b). We also found that maximal intensity of Ca^2+^ indicator signal was similar in both directly irradiated cells which underwent eventual cell death and adjacent cells which remained alive (Supplementary Fig. 4c). Therefore, we considered laser-induced ROS but not Ca^2+^ to be the primary cause of irreversible changes. To test this hypothesis, we used dihydroethidium (DHE), which is a ROS indicator that emits red fluorescence when oxidized. Only the irradiated cells with irreversible responses showed significant increases in DHE fluorescence (Fig. 3a and Supplementary Movie 3). In cells showing irreversible responses, DHE fluorescence was initiated from the vicinity of the laser irradiation region and propagated to the nucleus (Supplementary Fig. 5a,b). There was no increase in DHE fluorescence in cells with reversible responses or non-irradiated cells, whereas the cells with irreversible responses clearly showed increases in DHE fluorescence after laser stimulation (Fig. 3b). Based on these results, we presumed that there may be a specific threshold of ROS level that must be reached before cytotoxic effects occur.

In the presence of serum and sodium pyruvate within the culture medium, a higher laser energy was required to induce the same degree of cell death as in the experiment performed in the absence of serum and sodium pyruvate. We used culture medium free of serum and sodium pyruvate to exclude their antioxidant actions in the subsequent experiments. To test the effects of ROS on laser-induced dell death, cells were incubated with 25 mmol/L N-acetylcysteine (NAC), an antioxidant, for 30 min before optical stimulation. Treatment with NAC significantly reduced laser-induced cell death at an irradiation energy of 6.55 μJ, while the same treatment had no significant effect at an energy of 105.24 μJ (Supplementary Fig. 6a). These results indicate that laser-induced ROS are key molecules of cytotoxicity and that antioxidants can minimize laser-induced cytotoxic effects by eliminating laser-induced ROS.

We further investigated the signaling molecules involved in the laser-induced cell death process by targeting the caspase family and PARP-1, which are involved in apoptosis^16^,^17^. We used Boc-Asp(O-methyl)-fluoromethyl-ketone (BocD-fmk), which is a nonspecific caspase family inhibitor, and 3-Aminobenzamide (3AB), which is a PARP-1 inhibitor. Pharmacological inhibition of the caspase family and PARP-1 significantly reduced the cell death ratio at an irradiation energy of 6.55 μJ (Fig. 3c). However, the same treatment had no effect on cells exposed to femtosecond laser pulses at an energy of 25.61 μJ (Supplementary Fig. 6b). Cotreatment with BocD-fmk and 3AB enhanced the protective effects against femtosecond laser-induced cell death (Supplementary Fig. 6c). These results indicate that laser irradiation at an energy level of ~6 μJ induces cell death via the caspase and PARP-1 signaling pathway.

### Role of mitochondria in laser-induced cell death

Although femtosecond-pulsed laser was focused on a cytosolic area of less than 1 μm^2^, the laser-induced cell death process was accompanied by mitochondrial potential loss and fragmentation throughout the whole cytosol. To determine the role of mitochondria in the laser-induced cell death process, we used a mitochondrial permeability transition pore (mPTP) blocker, cyclosporine A (CysA), and genetic modification using small interfering RNAs (siRNAs) against mitochondrial dynamics-related genes, Drp1 and Mfn1. To block mPTP, HeLa cells were incubated with 5 mg/L CysA for 45 min before optical stimulation. CysA significantly reduced laser-induced cell death at an irradiation energy of 6.32 μJ (Supplementary Fig. 6d), while the same treatment had no effect when cells were exposed to laser pulses at a higher energy. Comparable results were obtained when cells were treated with antioxidant or chemical inhibitors. At laser energy levels of ~6 μJ, chemical inhibitors showed protective effects against laser-induced cell death, while the same treatment had little effect when cells were exposed to laser pulses at a higher energy level.

**Figure 4:**
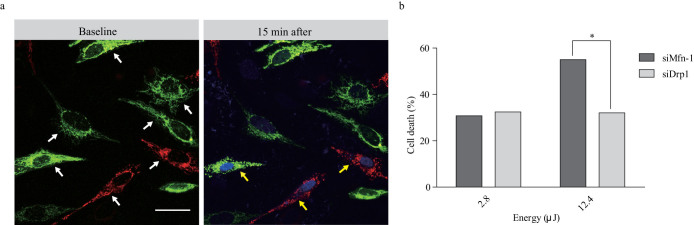
Analysis of mitochondrial dynamics via laser-induced intrinsic ROS. (a) Representative images of genetically modified mitochondrial dynamics and laser-induced cell death. Green fluorescence indicates mitochondrial fusion caused by siDrp1 treatment. Red fluorescence indicates mitochondrial fission caused by siMfn1 treatment. The white arrows indicate irradiated cells. The yellow arrows indicate cells that showed cell death after laser stimulation. (b) Laser-induced cell death rates are shown in relation to mitochondrial dynamics. The experiments revealed the cell death ratio caused by laser stimulation (*n* = 55 cells). *P < 0.05 (Chi-square test).

Mitochondrial dynamics affected intracellular ROS level, and fragmented mitochondria are more susceptible to ROS cascades than elongated mitochondria^18^. To determine effects of mitochondrial dynamics on laser-induced cell death, we modified the mitochondrial network using siDrp1 and siMfn1 (Supplementary Fig. 7a). Altering the Drp1 and Mfn1 proteins significantly changed the morphologies of mitochondria into elongated and fragmented forms, respectively (Supplementary Fig. 7b). To obtain a homogenous optical path and extracellular conditions, we co-plated two different genetically modified cells into the same dish 48 h after siRNA transfection together with mito-GFP and mito-DsRed, respectively (Fig. 4a). Down-regulation of Drp1 protected cells from laser-induced cell death; while inhibition of Mfn1 increased sensitivity against laser-induced cell death compared to scramble siRNA control (Supplementary Fig 7c, d). Although there were no significant differences in cell death ratio at an energy of 2.8 μJ (Fig. 4b), we found that cell death ratio was significantly reduced in cells with elongated mitochondria at an irradiation energy of 12.4 μJ. Taken together, the result of chemical inhibitor and genetic modification experiments indicated that femtosecond laser irradiation induced cell death in a mitochondria-dependent manner.

## Discussion

In the present study, we have demonstrated for the first time that brief irradiation of femtosecond laser pulses induce an apoptosis-like cell death in a mitochondria-dependent manner. Irradiation above a certain energy level generated ROS at the irradiated area, which were further amplified and then propagated through the whole cell ultimately becoming an irreversible response leading to cell death regardless of cell types. This is accompanied by phenotypic changes such as mitochondrial membrane potential loss, cell shrinkage and plasma membrane bleb formation.

Indeed, we demonstrated that mitochondrial function is essential for laser-induced cell death. Laser irradiation induced mitochondria-related changes, i.e., mitochondrial fragmentation and potential loss, ROS amplification, and activation of caspase and PARP-1 signaling. One possible mechanism for ROS amplification is ROS-induced ROS release (RIRR), which is a process that accelerates ROS production itself via the mitochondrial network^19^. mPTP inhibition and elongated mitochondria had a significantly reduced laser-induced cell death ratio. mPTP is the process of outer mitochondrial membrane permeabilization that releases of several proapoptotic proteins from the mitochondrial intermembrane space, including cytochrome c^17^. These data support that conclusion that laser-induced cell death depends on mitochondria.

The type of laser-induced cell death described here is distinct from apoptosis, necrosis, and autophagy. It shares some common morphological characteristics and cellular signaling pathways with apoptosis. While apoptosis exhibits cell death phenotypes several hours after the apoptosis-initiating stimulus^14^, cell death caused by femtosecond laser irradiation shows morphological changes within several minutes after laser irradiation. Necrosis is a form of caspase-independent cell death associated with plasma membrane rupture, and autophagy forms double membrane-enclosed vesicles^20^. However, laser-induced cell death does not show any morphological or chemical similarities with necrotic death or autophagy. An iron-depedent form of non-apoptotic cell death called ferroptosis was recently discovered. The mechanisms underlying ferroptosis remain unclear, but it is related to iron-dependent accumulation of intracellular ROS and mitochondrial damage^20^. In terms of intracellular ROS surge and mitochondrial damage, ferroptosis shows some similarities with laser-induced cell death. However, ferroptosis has been reported to occur regardless of caspase activation, while we found that caspase signaling is involved in laser-induced cell death. Although further investigations are required to define the type of laser-induced cell death process, it shares some common features with apoptosis and ferroptosis.

Previous studies have indicated that femtosecond laser irradiation can induce cell death by ROS production^9^,^12^ or plasma membrane disruption^21^. However, these studies focused on the effects of ultrashort-pulsed laser exposure on biological samples without considering intracellular events. In contrast, our results clearly showed that femtosecond laser pulses focused on less than 1 μm^2^ for several hundred microseconds are sufficient to trigger intracellular cell death signaling pathways via localized ROS production in the irradiated region. Thus, femtosecond laser pulses likely intervene in ROS amplification and downstream of ROS accumulation. These findings will be valuable for future studies of various ROS-mediated intracellular signaling pathways at the single-cell level.

In conclusion, we have shown that femtosecond laser irradiation induces apoptosis-like cell death in a mitochondria-dependent manner only in irradiated cells without any deleterious effects on adjacent cells. Femtosecond laser pulses produce excess intracellular ROS in cells, with an irreversible response, leading to mitochondrial damage and ultimately apoptosis-like cell death. Our chemical inhibitor experiments and mitochondrial dynamics experiments indicated protective effects of laser irradiation at moderate energy, but there were no protective effects at high energy. Further studies are required to elucidate why chemical inhibitors and genetic modifications did not show protective effects at high energy. Even though we also showed that laser-induced cell death is calcium-independent process, further studies are required to clarify roles of Ca^2+^ in laser-induced cell death. Although further studies are needed, we showed, for the first time, that femtosecond laser pulses intervene in the intrinsic cellular signaling pathway without chemical agents or genetic modification.

## Methods

### Cell Culture

HeLa cells were maintained in Dulbecco's Modified Eagle's Medium(DMEM, Gibco) supplemented with 10% heat-inactivated fetal bovine serum (FBS), 4500 mg/L D-glucose, L-glutamine, 110 mg/L sodium pyruvate, sodium bicarbonate, 1000 U/L penicillin, and 100 μg/mL streptomycin. For antioxidant drug experiments, HeLa cells were incubated for 12 h in DMEM complete medium without FBS and sodium pyruvate.

### Genetic modification

To investigate the relationship between mitochondrial dynamics and laser-induced ROS, proteins involved in the mitochondrial fusion and fission process (Mfn-1 and Drp1) were depleted using small interfering RNAs (siRNAs). The following RNA oligonucleotides were used: human Mfn1: 5′-GUGUAGAUUCUGGUAAUGA-3′, human Drp1: 5′-GAGGUUAUUGAACGACUCA-3′; negative control 5′-CCUACGCCAAUUUCGU-3′. The siRNAs were transiently transfected using Lipofectamine 2000 (Invitrogen) in accordance with the manufacturer's instructions. One day after transfection, media were changed and cells were grown for an additional 48 h before the experiments. To study the roles of mitochondrial morphology in laser-induced cell death, each sample was transiently cotransfected with target siRNA and mito-green fluorescent protein (Mito-GFP) or mito-red fluorescent protein (Mito-DsRed), respectively. Two different protein knockdown cells were co-plated into the same culture dishes to achieve the same optical and culture environmental conditions.

### Imaging

We used confocal and two-photon laser scanning microscopy (LSM510; Carl Zeiss) with femtosecond pulse laser (Chameleon; Coherent) and a water emersion objective lens (40×, 0.8 numerical aperture). For cellular imaging, we used a PDMI2 Open Perfusion Micro-Incubator (Harvard Apparatus) with the medium temperature maintained at 37°C and 5% CO_2_.

### Laser irradiation

The optical path and laser source for optical intervention were the same as those used in the two-photon fluorescence imaging. For the *in vitro* study, we selected a target region of <1 μm^2^ in the cytosol of a HeLa cell. The irradiation laser output power was fixed at 1 W, and the duration was changed from 1.96 to 196.83 μs to adjust the average stimulation energy. To confirm laser-induced cell death, we stained cells with cell impermeable indicators PI and DAPI at 15 min after laser stimulation and observed the fluorescence signal.

### Chemicals

For calcium staining, HeLa cells were loaded with 10 μmol/L of the acetoxymethyl form of Fluo-4 (Fluo-4AM; Sigma) at 37°C for 40 min in medium and then washed twice with medium. For mitochondrial staining, the cells were loaded with 200 nmol/L MitoTracker (Invitrogen) at 37°C for 5 min in medium and then washed twice with medium. To confirm cell death caused by laser stimulation, 1 mg/L propidium iodide (PI; Sigma-Aldrich) and 5 mg/L DAPI (Invitrogen) were added to complete DMEM during the imaging experiment. For plasma membrane staining, the cells were loaded with 5 mg/L CellMask (Invitrogen) at 37°C for 15 min in medium and then washed twice with medium. To measure laser-induced ROS generation, 10 μmol/L dihydroethidium (DHE; Invitrogen) was added to medium immediately before laser stimulation experiments. To study the mechanism of cell death, cells were treated with 10 mmol/L 3-aminobenzamide (3AB; Sigma-Aldrich), which is a poly (ADP-ribose) polymerase 1 (PARP-1) inhibitor, for 1 h before the experiments, and 20 μmol/L Boc-Asp(O-methyl)-fluoromethyl-ketone (BocD-fmk; BioVision), which is a caspase family inhibitor, was used for pretreatment for 30 min before the experiments. For antioxidant experiments, 25 mmol/L *N*-acetylcysteine (NAC; Sigma-Aldrich) was added to culture medium 30 min before the experiments. For mitochondrial permeability transition pore (mPTP) confirmation, cells were pretreated with 5 mg/L cyclosporine A (CysA; Sigma-Aldrich), which is an inhibitor of mPTP, 45 min before the experiments.

### Data analysis and statistical analysis

We used ImageJ for image processing and data quantification. Statistical analyses were performed using the GraphPad Prism software. Data are presented as means ± SEM. Statistical significance of differences was analyzed using Pearson's chi-squared test. In all analyses, *P* < 0.05 was taken to indicate statistical significance.

## Author Contributions

J.Y. designed and performed experiments, analyzed data and wrote the paper; S.R. performed genetic modification experiments; S.L. performed and analyzed mPTP experiment; C.C. designed experiments, managed the project and wrote the paper. All the authors read and reviewed the manuscript, agreed that it was ready for publication and accepted responsibility for its contents.

## Supplementary Material

Supplementary Information Supplementary Movie 1

Supplementary Information Supplementary Movie 2

Supplementary Information Supplementary Movie 3

Supplementary Information Supplementary figures

## References

[b1] Yoon J., Park J., Choi M., Jong Choi W. & Choi C. Application of femtosecond-pulsed lasers for direct optical manipulation of biological functions. Ann Phys (Berlin) 525, 205–214 (2012).

[b2] Choi M., Yoon J. & Choi C. Label-free optical control of arterial contraction. J Biomed Opt 15, 015006-1-6 (2010). 20210446 10.1117/1.3316404

[b3] Yoon J., Choi M., Ku T., Choi W. J. & Choi C. Optical induction of muscle contraction at the tissue scale through intrinsic cellular amplifiers. J Biophotonics 7, 597–606 (2014). 23650149 10.1002/jbio.201200246

[b4] Choi M., Ku T., Chong K., Yoon J. & Choi C. Minimally invasive molecular delivery into the brain using optical modulation of vascular permeability. Proc Natl Acad Sci USA 108, 9256–9261 (2011). 21576460 10.1073/pnas.1018790108 PMC3107279

[b5] Choi M., Yoon J., Ku T., Choi K. & Choi C. Label-free optical activation of astrocyte in vivo. J Biomed Opt 16, 075003-1-5 (2011). 21806260 10.1117/1.3600774

[b6] Hirase H., Nikolenko V., Goldberg J. H. & Yuste R. Multiphoton stimulation of neurons. Dev Neurobiol 51, 237–247 (2002). 10.1002/neu.10056 11984845

[b7] Antkowiak M., Torres-Mapa M. L., Stevenson D. J., Dholakia K. & Gunn-Moore F. J. Femtosecond optical transfection of individual mammalian cells. Nat Protoc 8, 1216–1233 (2013). 23722260 10.1038/nprot.2013.071

[b8] Vogel A., Noack J., Hüttman G. & Paltauf G. Mechanisms of femtosecond laser nanosurgery of cells and tissues. Appl Phys B 81, 1015–1047 (2005).

[b9] Tirlapur U. K., König K., Peuckert C., Krieg R. & Halbhuber K.-J. Femtosecond near-infrared laser pulses elicit generation of reactive oxygen species in mammalian cells leading to apoptosis-like death. Exp Cell Res 263, 88–97 (2001). 11161708 10.1006/excr.2000.5082

[b10] Thannickal V. J. & Fanburg B. L. Reactive oxygen species in cell signaling. Am J Physiol Lung. Cell Mol Physiol 279, L1005–L1028 (2000). 11076791 10.1152/ajplung.2000.279.6.L1005

[b11] Orrenius S., Gogvadze V. & Zhivotovsky B. Mitochondrial oxidative stress: implications for cell death. Annu Rev Pharmacol Toxicol 47, 143–183 (2007). 17029566 10.1146/annurev.pharmtox.47.120505.105122

[b12] Uchugonova A. *et al.* Optical knock out of stem cells with extremely ultrashort femtosecond laser pulses. Journal of Biophotonics 1, 463–469 (2008). 19343672 10.1002/jbio.200810047

[b13] Carafoli E. Intracellular calcium homeostasis. Annu Rev Biochem 56, 395–433 (1987). 3304139 10.1146/annurev.bi.56.070187.002143

[b14] Elmore S. Apoptosis: a review of programmed cell death. Toxicol Pathol 35, 495–516 (2007). 17562483 10.1080/01926230701320337 PMC2117903

[b15] Kass G. & Orrenius S. Calcium signaling and cytotoxicity. Environ Health Persp 107, 25–35 (1999). 10.1289/ehp.99107s125 PMC1566353 10229704

[b16] Virág L., Robaszkiewicz A., Rodriguez-Vargas J. M. & Oliver F. J. Poly (ADP-ribose) signaling in cell death. JMAM 34, 1153–1167 (2013). 10.1016/j.mam.2013.01.007 23416893

[b17] Orrenius S. Reactive oxygen species in mitochondria-mediated cell death. Drug Metab Rev 39, 443–455 (2007). 17786631 10.1080/03602530701468516

[b18] Wang X. *et al.* Amyloid-beta overproduction causes abnormal mitochondrial dynamics via differential modulation of mitochondrial fission/fusion proteins. Proc Natl Acad Sci USA 105, 19318–19323 (2008). 19050078 10.1073/pnas.0804871105 PMC2614759

[b19] Park J., Lee J. & Choi C. Mitochondrial network determines intracellular ROS dynamics and sensitivity to oxidative stress through switching inter-mitochondrial messengers. PloS one 6, e23211 (2011). 21829717 10.1371/journal.pone.0023211 PMC3150422

[b20] Dixon S. J. *et al.* Ferroptosis: an iron-dependent form of nonapoptotic cell death. Cell 149, 1060–1072 (2012). 22632970 10.1016/j.cell.2012.03.042 PMC3367386

[b21] Prentice P., Cuschieri A., Dholakia K., Prausnitz M. & Campbell P. Membrane disruption by optically controlled microbubble cavitation. Nat Phys 1, 107–110 (2005).

