# Supplementary material for: Cytosolic Irradiation of Femtosecond Laser Induces Mitochondria-dependent Apoptosis-like Cell Death via Intrinsic Reactive Oxygen Cascades
Source: Sci Rep. 2015 Feb 4;5:8231. doi: 10.1038/srep08231 (PMC4316155; doi:10.1038/srep08231)

Supporting Information

Cytosolic Irradiation of Femtosecond Laser Induces Mitochondria-dependent Apoptosis-like Cell Death via Intrinsic Reactive Oxygen Cascades

Jonghee Yoon[1,2], Seung-wook Ryu[1,3], Seunghee Lee[1] & Chulhee Choi[1,2,3*]

[1]Department of Bio and Brain Engineering, KAIST, Daejeon, Korea

[2]KAIST Institute for Optical Science and Technology, KAIST, Daejeon, Korea

[3]KAIST Institute for the BioCentury, KAIST, Daejeon, Korea

Supplementary Figure 1. Laser-induced mitochondrial fragmentation. (**a**) Minimal overlap between punctuated Ca^{2+} fluorescence and mitochondrial fragmentation. Green fluorescence indicates intracellular Ca^{2+} and red fluorescence indicates mitochondria. The square with white lines at the bottom right is a higher magnification view of the square with white dashed lines. The white spot indicates the region of laser irradiation. Scale bar, 20 µm. (**b**) Temporal dynamics of laser-induced mitochondrial fragmentation. White arrows indicate the region of mitochondrial fragmentation. The white spot indicates the region of laser irradiation. Scale bar, 20 µm.

(**c**) Quantification of mitochondrial potential through variation in MitoTracker in both reversible and irreversible responses. Red fluorescence. The red arrow indicates laser irradiation time.

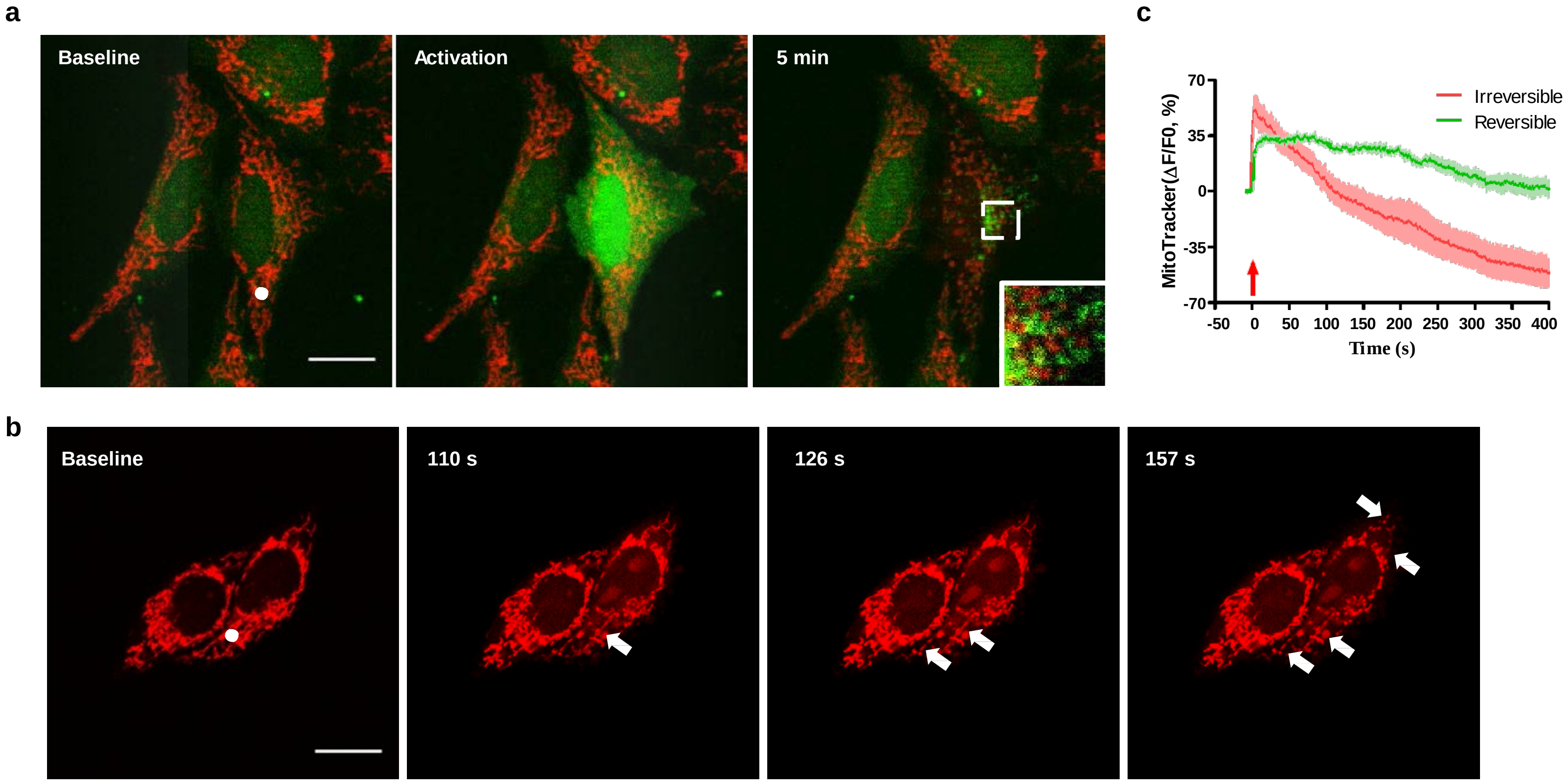

Supplementary Figure 2. Long-term observation of laser-induced specific apoptosis-like cell death by phase microscopy. (**a, b**) Arrows indicate laser-irradiated cells. The yellow arrow indicates the reversible response, and the red arrow indicates the irreversible response. The numbers above each image indicate the time after la ser stimulation.

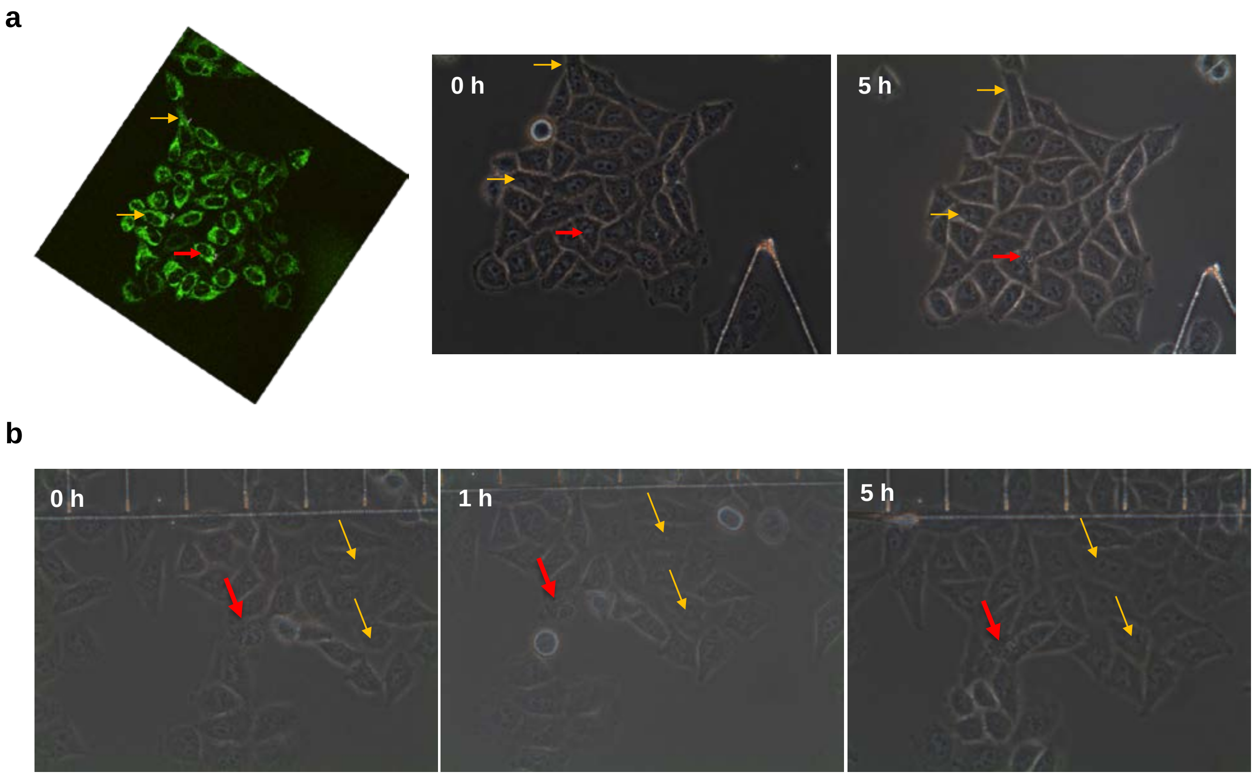

Supplementary Figure 3. Laser-induced cell death in various cell types. The white arrows indicate laser-irradiated cells. Red fluorescence indicates mitochondria s tained with MitoTracker Red and green fluorescence indicates intracellular Ca^{2+} ions stained with Fluo4-am. Scale bar, 50 μm.

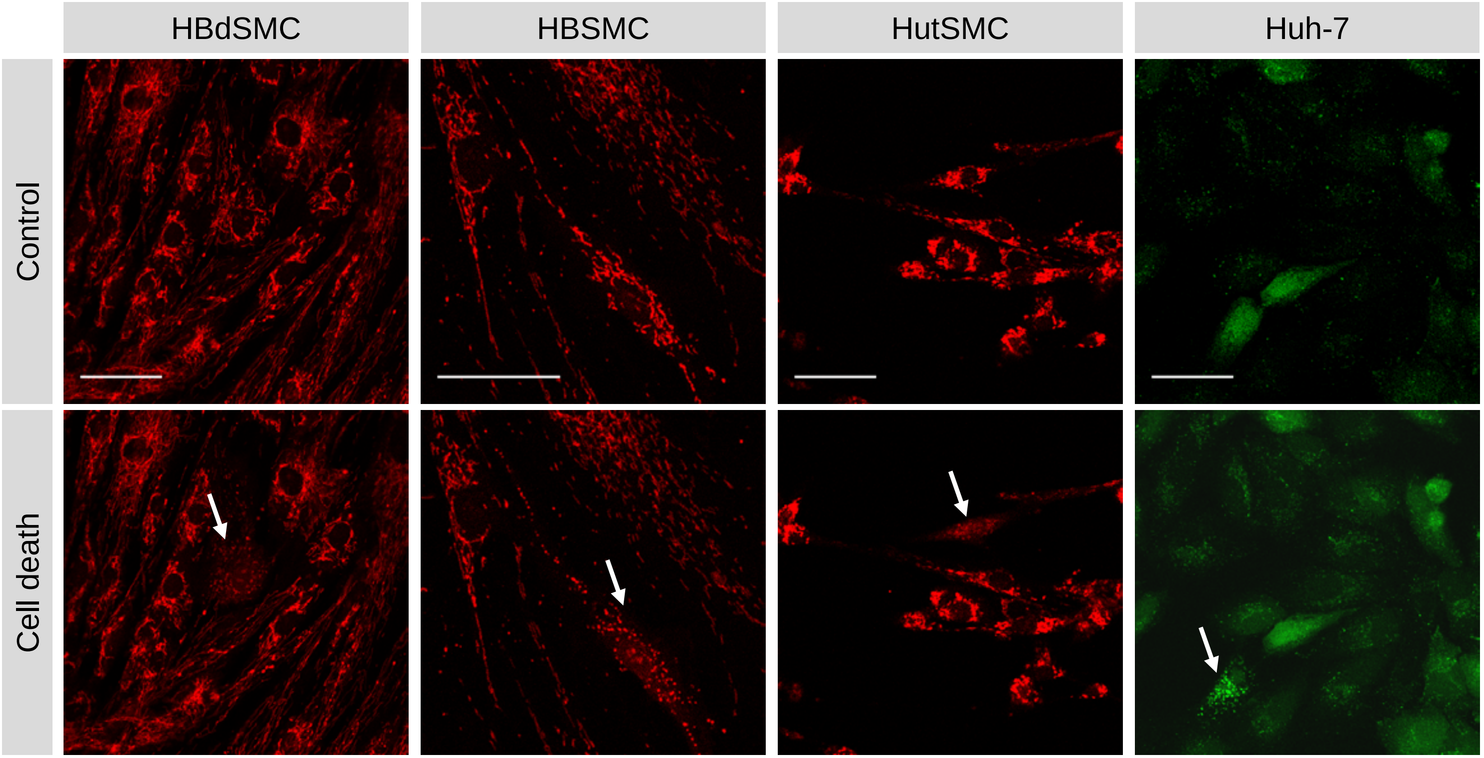

Supplementary Figure 4. Ca^{2+}-independent laser-induced cell death. (**a**) Temporal laser-induced Ca^{2+} dynamics with ryanodine receptor inhibition using the antagonist, ryanodine. The white circle indicates the region of laser irradiation. Scale bar, 20 μm. (**b**) Quantification of laser-induced Ca^{2+} dynamics in the irradiated cell in (a) and ryanodine-free cells. The red arrow indicates laser irradiation time. (n = 5) ***$P \leq 0.001$ (unpaired t-test). (**c**) Maximum Ca^{2+} indicator signals of irrdiated cellw which occure cell death and adjacent cells which are alive and increase intracellular Ca^{2+} level. (n = 6)

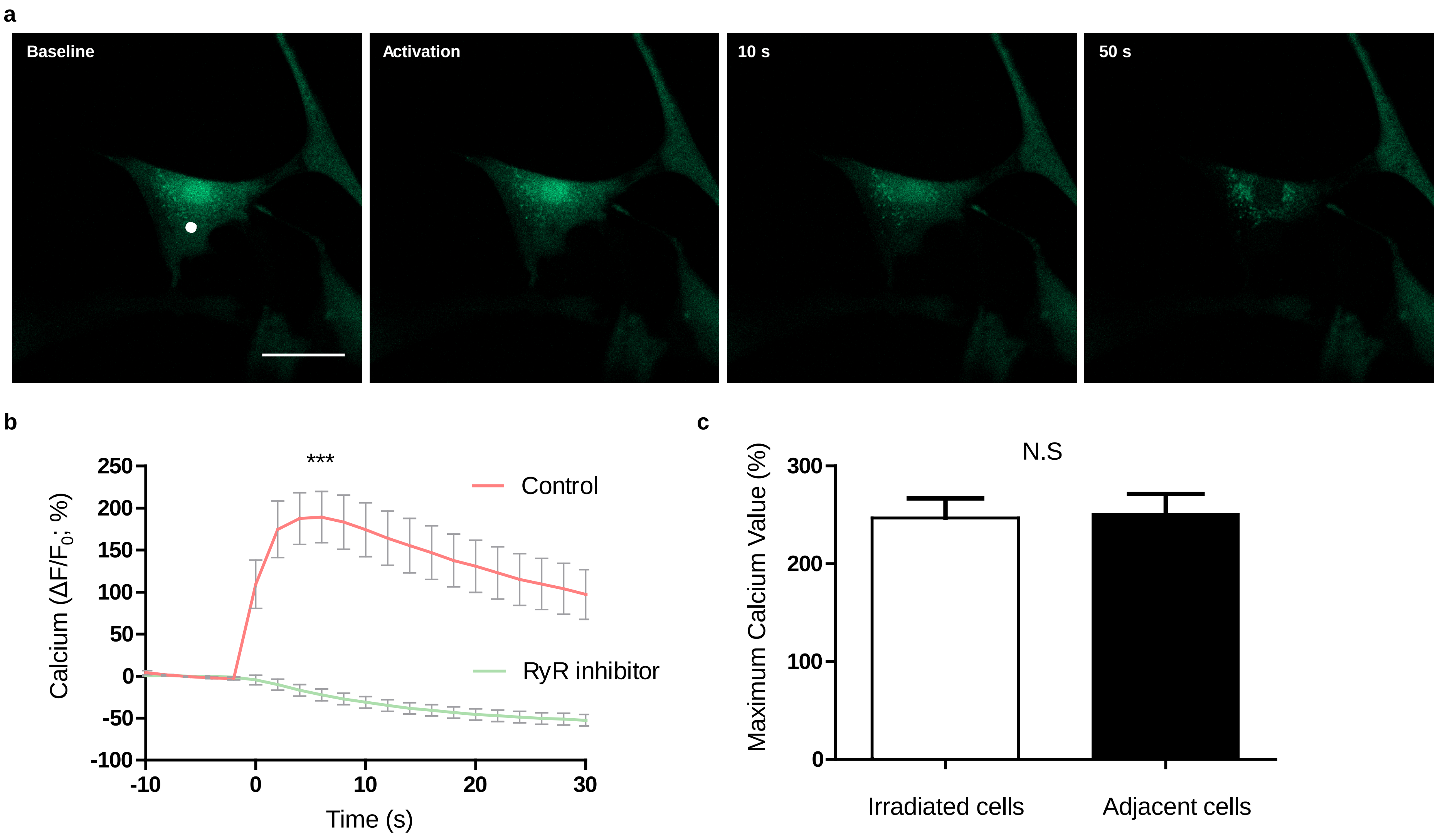

Supplementary Figure 5. Propagation of laser-induced ROS from the vicinity of the focal region to the nucleus. (**a**) Generation of intrinsic ROS via laser stimulation. Red fluorescence indicates endogenous ROS stained with DHE. Green fluorescence indicates mitochondria. The red spot indicates the region of laser irradiation. Scale bar, 20 μm. (**b**) Line scan image of the white arrow in (a) demonstrating laser-induced ROS propagation from the vicinity of the focal region to the nucleus. Laser-induced intrinsic ROS started to propagate to the lateral area. The white spot indicates the region of laser irradiation and time.

a

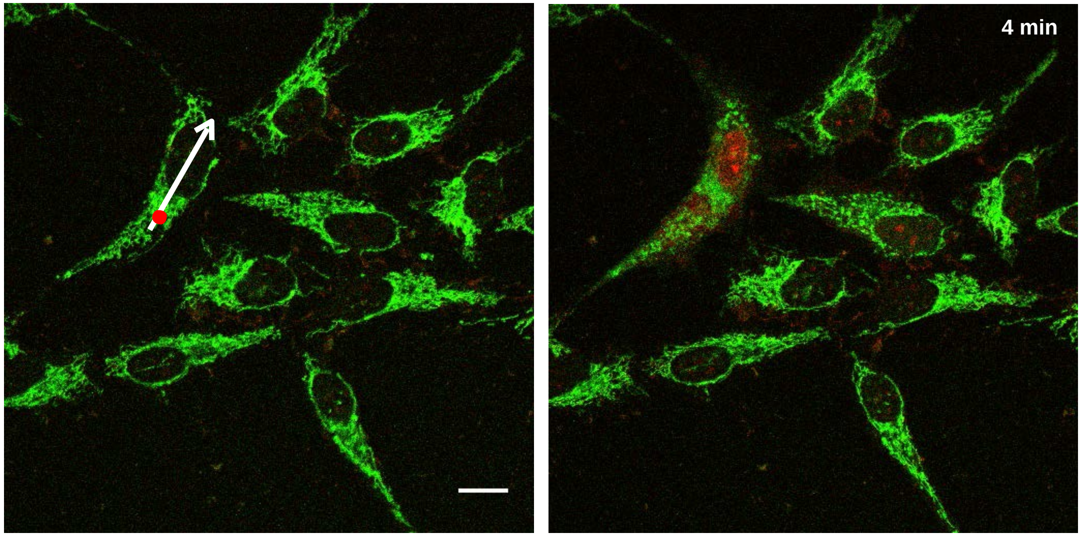

b

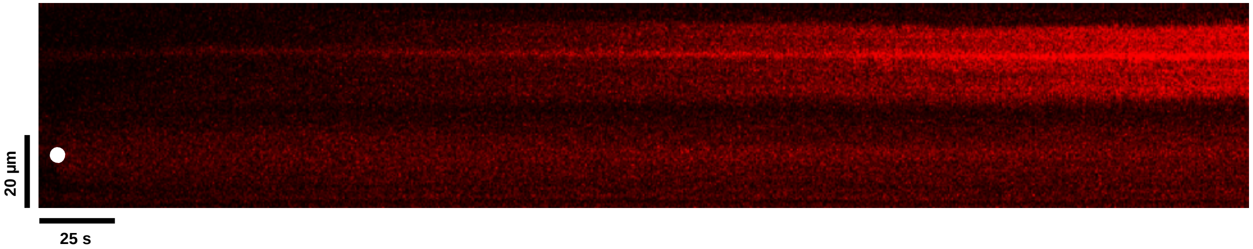

Supplementary Figure 6. Cellular signaling involved in laser-induced cell death. (**a**) Antioxidant effect against laser irradiation at 105.24 μJ. (**b**) Effects of 3AB (PARP inhibitor) and BocD-fmk (caspase inhibitor) on laser irradiation at 25.61 μJ. (*n* ≥ 35 cells). (**c**) Synergistic effects of 3AB and BocD-fmk on laser-induced cell death. The laser irradiation energy was 6.39 μJ. (*n* ≥ 100 cells). (**d**) Effect of cyclosporine A against laser-induced cell death. HeLa cells were treated with CysA (mPTP inhibitor, 5 mg/L). (*n* ≥ 100 cells) *$P \leq 0.05$, **$P \leq 0.01$, ***$P \leq 0.001$ (Chi-square test).

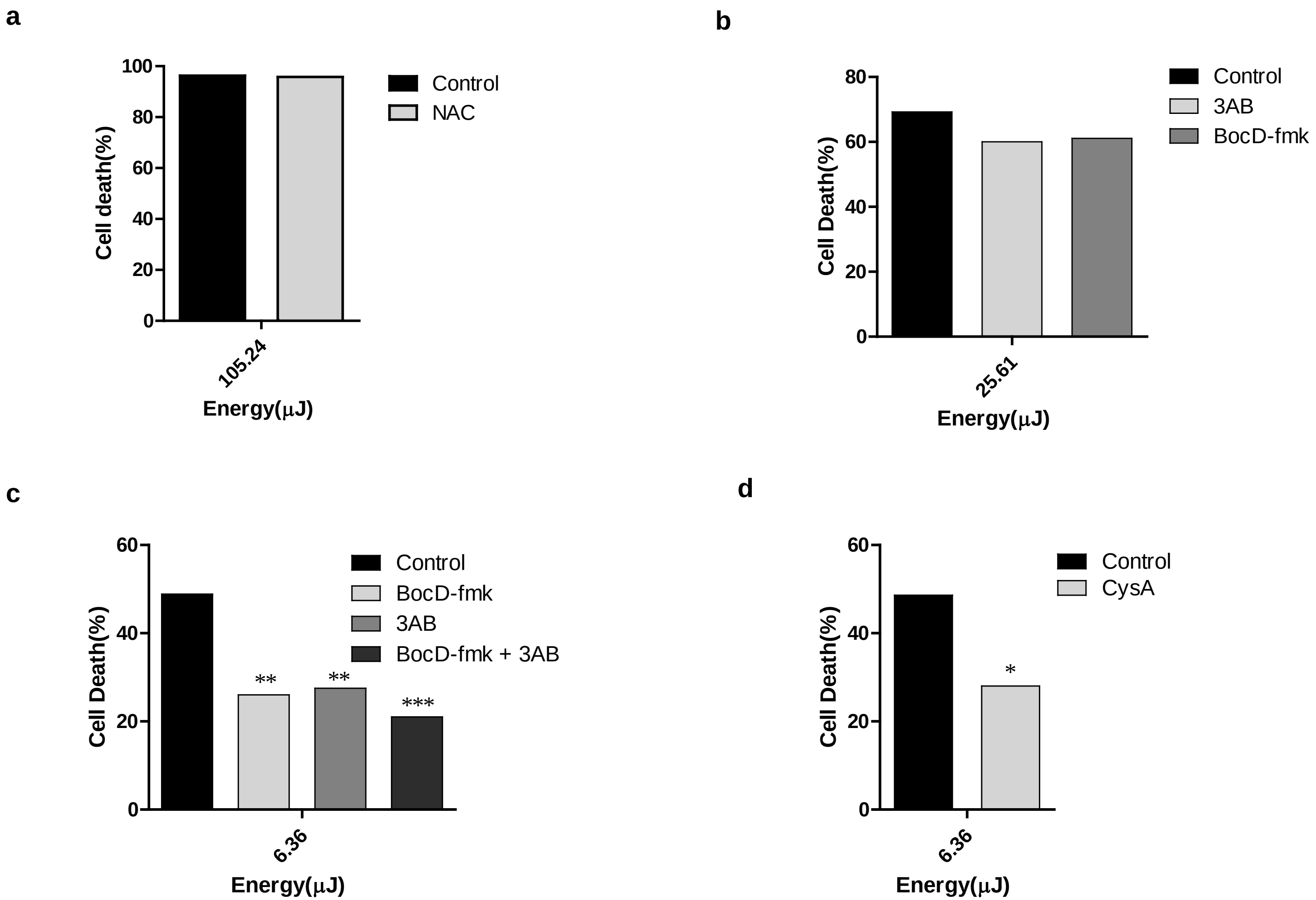

Supplementary Figure 7. Genetically modified mitochondrial morphology. (**a**) Real-time PCR evaluated levels of Drp1 and Mfn1 mRNA. Data are the mean ± S D of two experiments. *** p< 0.001. (**b**) Altered mitochondrial morphology throughout siControl, siDrp1, and siMfn1 treatment. Scale bar, 20 µm. (**c, d**) Effects of siRNAs against laser-induced cell death (n ≥ 20 cells).

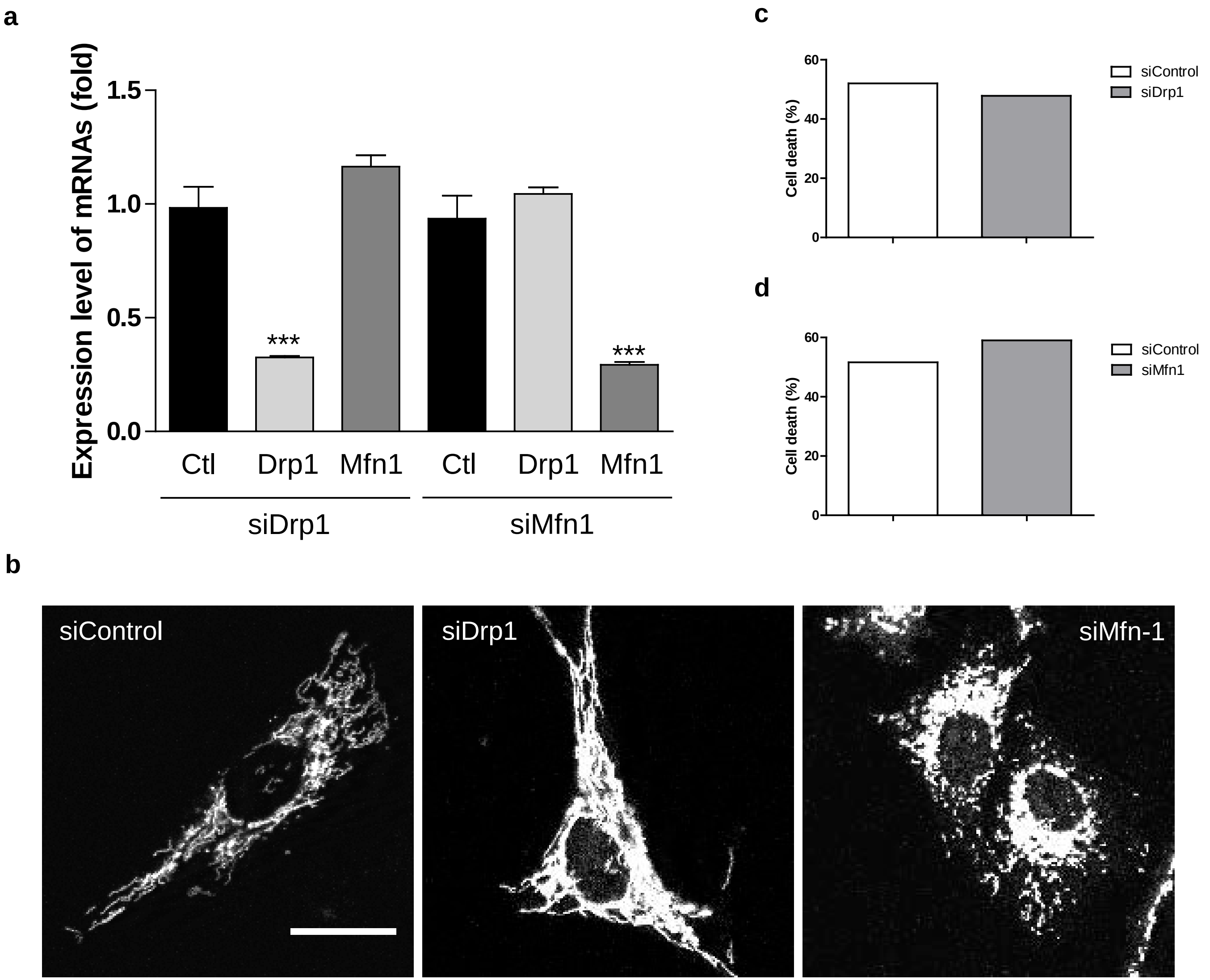

Supplementary Movie 1. Laser-induced mitochondrial fragmentation. Green fluorescence indicates intracellular Ca^{2+} stained by Fluo4-AM. Red fluorescence i ndicates mitochondria stained by MitoTracker Red. Scale bar, 20 μm.

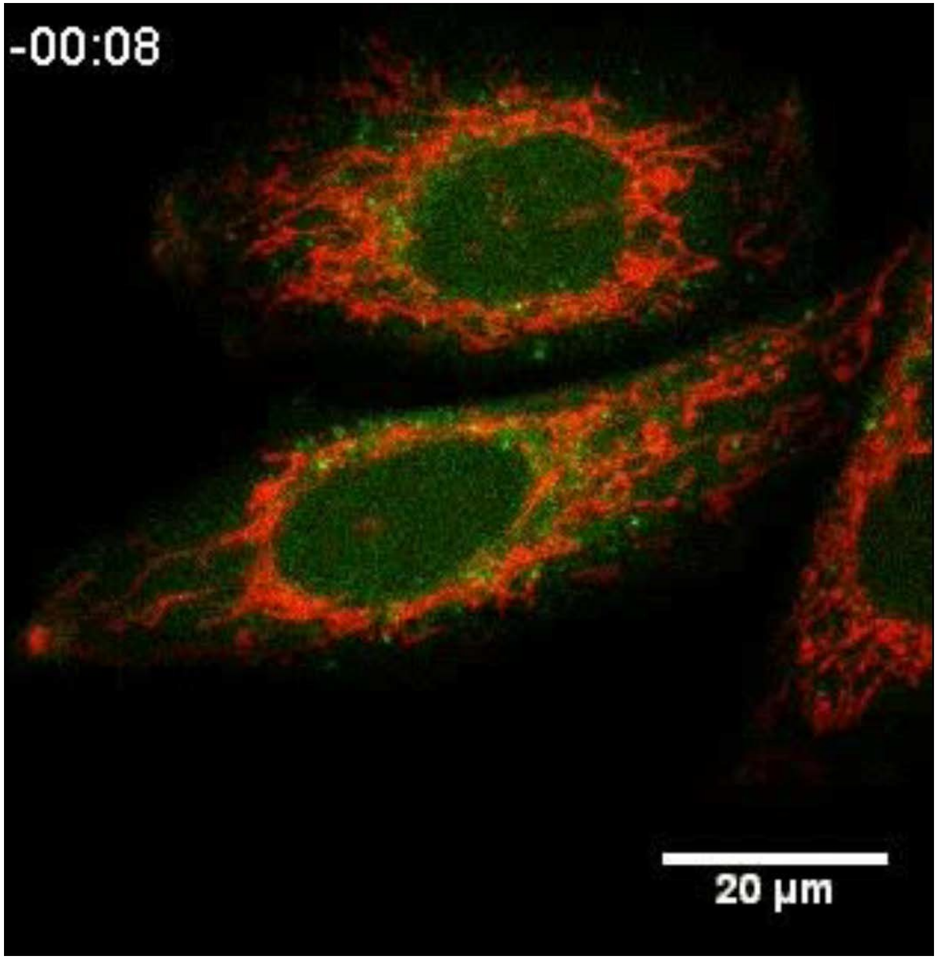

Supplementary Movie 2. Rapid plasma membrane retraction in the irradiated cells with the irreversible response. Changes in plasma membrane staining with CellMask. Green fluorescence indicates basal plasma membrane boundary and red fluorescence indicates changed plasma membrane boundary. Scale bar, 20 μm.

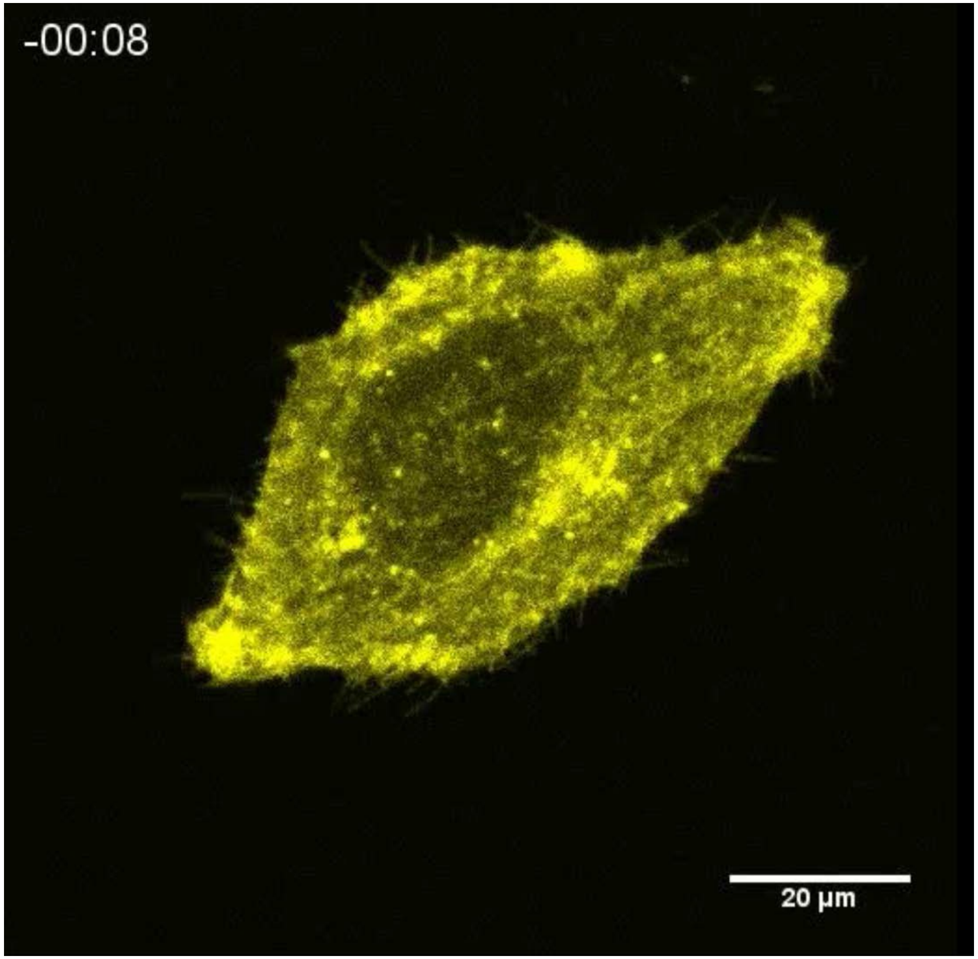

Supplementary Movie 3. Femtosecond laser pulses produce intrinsic ROS in the irradiated cells with irreversible response. Green fluorescence indicates mitochondria stained with MitoTracker Green. Red fluorescence indicates ROS generation stained with DHE. Scale bar, 50 μm.

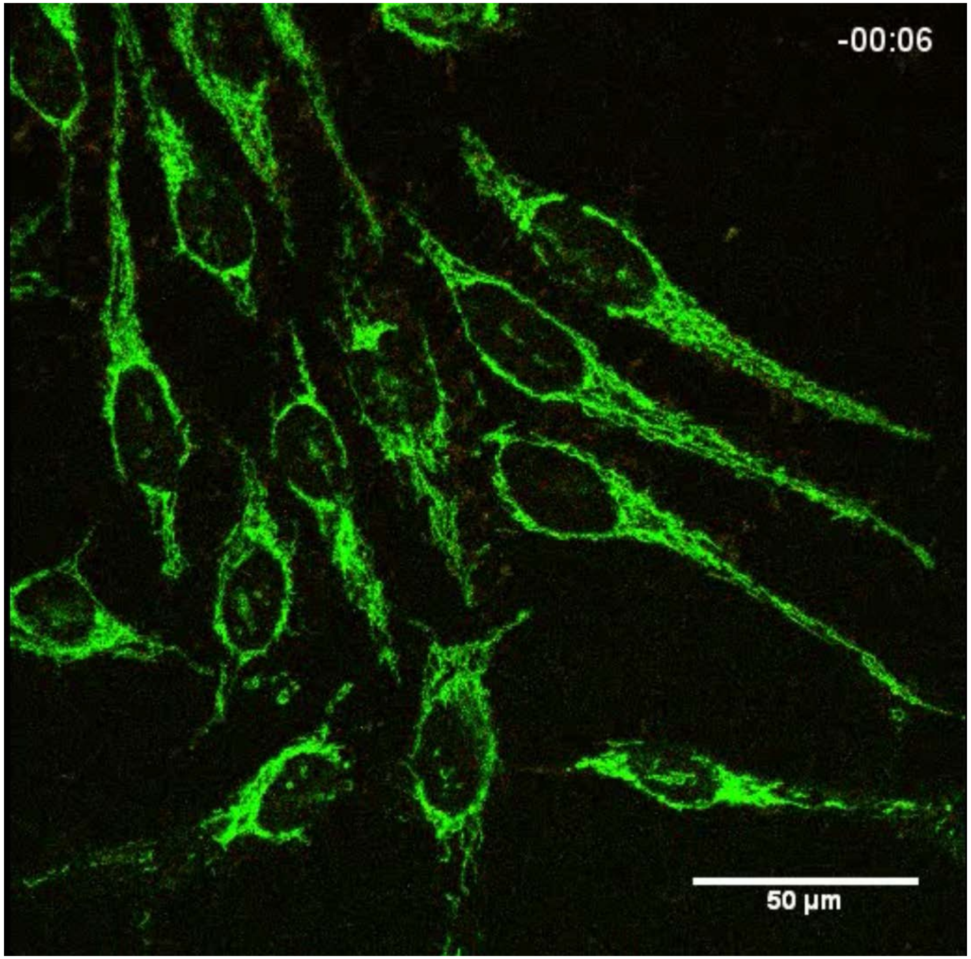

Supplement: Supplementary Information — Supplementary figures [file srep08231-s4.pdf]
